# Baicalin Decreases the LPS-Induced Intestine Inflammatory Responses by ROS/p-ERK/p-P38 Signal Pathways In Vivo and In Vitro

**DOI:** 10.3390/biomedicines13020251

**Published:** 2025-01-21

**Authors:** Xinyi Sun, Mengru Guo, He Su, Mei Liang, Huining Wu, Linlu Zhao, Jin Zhang, Jieyi He, Yanhong Yong, Zhichao Yu, Xingbin Ma, Xianghong Ju, Xiaoxi Liu

**Affiliations:** 1 Department of Veterinary Medicine, College of Coastal Agricultural Sciences, Guangdong Ocean University, Zhanjiang 524088, China; sy111012@163.com (X.S.);; 2 The Second Clinical Medical College of Guangzhou University of Chinese Medicine, Guangzhou 510120, China

**Keywords:** baicalin, ROS/p-ERK/p-P38, intestinal inflammation, IPEC-J2 cells, *mice*

## Abstract

**Background:** This study aimed to investigate the role of ROS/MAPK signaling pathways and the effects of baicalin in LPS-induced inflammatory responses in *mice* and porcine intestinal epithelial cells (IPEC-J2). **Methods**: In vivo, 18 male C57BL/6J *mice* were randomly divided into three groups (*n* = 6): control, LPS (3.5 mg/kg LPS administered intraperitoneally [ip] on day 7), and baicalin (200 mg/kg orally for 7 days, with LPS ip on day 7). On day 8, *mice* were sacrificed, and jejunal tissues were collected for H&E staining. ROS levels in serum and cytokine protein expressions (TNF-α and IL-6) in the jejunum were measured via ELISA, while intestinal MAPK proteins were analyzed using Western blotting. In vitro, the study involved two experimental setups: NAC (a ROS scavenger) and baicalin. For the NAC experiment, IPEC-J2 cells were divided into three groups: control, LPS, and NAC. In the LPS group, cells were treated with LPS (40 μg/mL) for 1 h. In the NAC group, cells were pretreated with NAC prior to LPS exposure. For the baicalin experiment, IPEC-J2 cells were divided into five groups: control, LPS, and baicalin at low (10 μM), medium (20 μM), and high (40 μM) doses. Cells were pretreated with baicalin for 24 h before LPS exposure. ROS/LDH levels and cytokine expressions in the supernatant were determined via ELISA, and MAPK protein expressions were assessed using Western blotting. **Results:** In vivo, LPS-induced oxidative stress and inflammatory responses in the intestine, reduced the villus height-to-crypt ratio, and significantly increased protein expressions of p-ERK, p-P38, JNK, and p-JNK (*p* < 0.05). Baicalin treatment significantly inhibited serum ROS levels (*p* < 0.01), reduced jejunal cytokine expressions (TNF-α and IL-6, *p* < 0.05), improved intestinal structural damage, and decreased p-ERK, p-P38, and p-JNK protein expressions (*p* < 0.05). In vitro, NAC significantly reduced ROS levels (*p* < 0.01), cytokine expressions (TNF-α and IL-6), and MAPK activation (ERK, JNK, P38, and their phosphorylated forms, *p* < 0.05). Baicalin also significantly decreased ROS (*p* < 0.05), TNF-α (*p* < 0.05), IL-6 (*p* < 0.05), and MAPK protein expressions (ERK, p-ERK, and p-P38, *p* < 0.05). Molecular docking demonstrated that baicalin effectively bound to ERK and P38 proteins. **Conclusions:** Baicalin mitigated LPS-induced inflammatory responses via the ROS/p-ERK/p-P38 signaling pathway in vivo and in vitro.

## 1. Introduction

The weaning process induced a series of stress reactions in *piglets*, including diarrhea, stunted growth, and reduced immunity [1]. Weaning resulted in impaired intestinal integrity, an inflammatory response, and oxidative stress, ultimately leading to impaired nutrient absorption [2,3,4]. Improving intestinal health was imperative to optimize the nutritional digestibility and disease resistance of weaned *piglets*. Inflammation represented the fundamental mechanism by which the immune system identified and responded to infections, pathogenic microorganisms, trauma, allergic reactions, and other external stimuli [5]. A variety of inflammatory cytokines, including tumor necrosis factor α (TNF-α) and interleukin 6 (IL-6), were released by tissue cells in varying quantities. Oxidative stress was defined as an imbalance between the oxidative and antioxidant systems of cells and tissues, resulting from excessive production of reactive oxygen species (ROS), which were generated by mitochondrial respiration [6]. However, N-acetyl-L-cysteine (NAC) directly neutralized ROS, exerting antioxidant effects. Mitogen-activated protein kinases (MAPKs) were activated by various extracellular signals and stimuli, including extracellular signal-regulated protein kinase (ERK), c-Jun NH2-terminal kinase (JNK), and p38-MAPK [7]. These pathways played pivotal roles in regulating the expression of inflammatory cytokines [8], which also occurred in response to oxidative stress [9,10]. ROS generated in oxidative reactions regulated the pro-inflammatory response in treating inflammation-associated degenerative neurological diseases in microglial cells [11]. The intricate and complex relationship between inflammation and oxidative stress suggested the existence of a potential pathway connecting the inflammatory response and oxidative stress.

Baicalin, a flavonoid derived from the root of Scutellaria baicalensis, possessed significant anti-inflammatory properties in the context of oral inflammatory diseases, liver disease, and hepatic injury [12]. In China, baicalin was incorporated into animal feed over extended periods to enhance the growth performance of broilers and weaned *piglets*, in compliance with government regulations, due to its low toxicity and high biological activity [13]. Long-term dietary administration of baicalin in postpartum cows was demonstrated to enhance economic value by stimulating milk production [14]. In a *murine* model of acute lung injury, baicalin alleviated lung inflammation induced by lipopolysaccharide (LPS) or complement alternative pathway activation [15,16]. Furthermore, in a murine model of diethyl nitrosamine-induced liver cirrhosis, baicalin protected *mice* by inhibiting oxidative stress and inflammation [17]. Our previous research demonstrated that baicalin reduced inflammatory responses and oxidative stress in LPS-stimulated porcine intestinal epithelial cells (IPEC-J2) [18]. However, the mechanisms through which baicalin regulated inflammatory responses and oxidative stress remained incompletely understood. A scientific hypothesis suggested that ROS/MAPK signaling pathways regulated by baicalin might serve as a link between inflammatory responses and oxidative stress in LPS-induced intestinal inflammation. This hypothesis, however, was yet to be fully elucidated in the present study.

## 2. Methods

### 2.1. Experiment Material

Baicalin was purchased from Hubei Biotechnology Co., Ltd. ((Wuhan, China)), with a purity > 95%. IPEC-J2 cells were obtained from BeNa Culture Collection ((Beijing, China)), while N-acetyl-L-cysteine (NAC) was procured from BestBio (BB-47059, Shanghai, China). Lipopolysaccharide (LPS) was purchased from Sigma (L2880).

### 2.2. Grouping and Processing

Eighteen male C57BL/6J *mice* (7–8 weeks old, weighing 25–27 g) were selected for the study and maintained under pathogen-free conditions at a room temperature of 25 ± 2 °C, relative humidity of 50 ± 15%, and a 12 h/12 h dark-light cycle in the Animal House. The groups are outlined in Table 1. All animal care and experimental procedures adhered to protocols approved by the Animal Care and Use Committee of Guangdong Ocean University School of Medicine (Approval Number: 2022-scuec-021) and complied with the Guidelines on Animal Welfare of Guangdong Ocean University School of Medicine.

Porcine intestinal epithelial cells (IPEC-J2) were cultured in Dulbecco’s Modified Eagle’s Medium (DMEM/F-12 (1:1) basic, C11330500BT, Gibco, Los Angeles, CA, USA) supplemented with 10% fetal bovine serum (FBS, Z7186FBS, ZETA, Paris, France), 50 IU/mL penicillin, and 50 mg/mL streptomycin (Penicillin-Streptomycin, 15140-122, Gibco, Los Angeles, CA, USA) at 37 °C with 5% CO_2_. The medium was changed 48 h after the initial cell culture. When the cells reached 90% confluence, IPEC-J2 cells were seeded into 6-well plates. The grouping details are provided in Table 2 and Table 3. IPEC-J2 cells were stimulated with 40 μg/mL LPS (L2880, Sigma, St. Louis, MO, USA) for 1 h after pretreatment with NAC for 1 h and baicalin for 24 h. In the NAC group, the NAC stock solution was diluted 1:500, as per the manufacturer’s instructions, and used to treat IPEC-J2 cells. Each experiment was conducted in triplicate.

### 2.3. CCK-8 Assay to Measure the Appropriate Concentration of LPS

To select the optimal concentration and duration of LPS treatment, equal numbers of IPEC-J2 cells were seeded in 96-well plates (Wuxi NEST Life Technology Co., Ltd., Wuxi, Jiangsu, China) and cultured in DMEM/F-12 medium containing 10% FBS. Once the cells reached 95% confluence, they were treated with different concentrations of LPS (10–80 μg/mL) for 1, 3, and 6 h. CCK-8 reagent (CK001, Beijing HuameiShengke, Haidian, Beijing, China) was then added to each well (10 μL per well), and the cells were incubated at 37 °C with 5% CO_2_ for 1 h. The absorbance at 450 nm was measured using a microplate reader (BioTek, Winooski, VT, USA).

### 2.4. Histopathology of the Jejunum

The jejunum was preserved in 4% paraformaldehyde for histopathological evaluation. The samples were embedded in paraffin following the standard procedure, sectioned, and stained with hematoxylin and eosin (H&E). Histopathological changes were observed and evaluated under a light microscope (Nikon, Tokyo, Japan).

### 2.5. Enzyme-Linked Immunosorbent Assay (ELISA)

The tissue sample was removed from the −80 °C freezer and placed on ice. A certain amount of tissue was weighed and transferred to a pre-cooled centrifuge tube. An appropriate amount of protein lysate (RIPA buffer with protease inhibitor at a 100:1 ratio) was added, and the tube was placed in a grinder for tissue homogenization. After grinding, the tube was kept on ice or in a 4 °C low-temperature metal bath for 30 min. Following lysis, the sample was centrifuged at 12,000 rpm for 30 min at 4 °C. For IPEC-J2 cells cultured in 6-well plates (Wuxi NEST Life Technology Co., Ltd.) to 95% confluence, the cells were washed twice with phosphate-buffered saline (PBS) and treated with different concentrations of baicalin (10–40 μM) for 24 h. The cells were then washed twice with PBS and activated with LPS (40 μg/mL) for 1 h. The supernatant was collected and centrifuged at 3000 rpm for 10 min. For the NAC treatment group, the cell treatment method was the same as described above. After PBS was added, the cells were lysed, and the supernatant was collected for ROS detection after centrifugation at 4000 rpm for 10 min. TNF-α (MM-0132M2), IL-6 (MM-0163M2), ROS (MM-3284901), and LDH (MM-36393O1, Jiangsu Enzyme Immune Industrial Co., Ltd., Yancheng, Jiangsu, China) levels were measured using ELISA kits according to the manufacturer’s instructions. Normalization analysis was performed based on cellular protein concentration.

### 2.6. Western Blot

RIPA lysis buffer (Beyotime, P0013B, Shanghai, China) was used to extract proteins from jejunal tissue and IPEC-J2 cells. The BCA protein detection kit (Beyotime, KGP1100, China) was used to quantify protein concentration. Proteins (20 μg/sample) were separated by SDS-PAGE (Tricine-SDS-PAGE gel kit, Cwbio, Beijing, China), transferred to polyvinylidene fluoride (PVDF) membranes (Sigma-Aldrich, ISEQ00010, St. Louis, MO, USA), and blocked in rapid plaque blocking buffer (Beyotime, Shanghai, China) for 30 min. The membrane was incubated with primary antibodies and hybridized with specific secondary antibodies. The following antibodies were used: p38 (1:1000, #8690, Cell Signaling Technology, Danvers, MA, USA), p-p38 (1:1000, #9211, Cell Signaling Technology, Danvers, MA, USA), ERK (1:1000, #4695, Cell Signaling Technology, Danvers, MA, USA), p-ERK (1:1000, #4370, Cell Signaling Technology, Danvers, MA, USA), JNK (1:1000, #9252, Cell Signaling Technology, Danvers, MA, USA), p-JNK (1:1000, #9251, Cell Signaling Technology, Danvers, MA, USA), β-actin (1:1000, #4970, Cell Signaling Technology, Danvers, MA, USA), and secondary antibody (1:1000, Goat Anti-Rabbit IgG (H+L), HRP Conjugate, HS101-01, TransGen Biotech, Beijing, China). The ECL detection system (Tanon, Shanghai, China) was used to visualize the imprints, and protein quantification was performed using the Image J 1.52a/Java 1.8.0_112 (64-bit) analyzer (NIH, Bethesda, Rockville, MD, USA).

### 2.7. Molecular Docking

The structures of ERK (PDB ID: 2zoq) and p38 (PDB ID: 1bl7), retrieved from the RCSB PDB database (https://www.rcsb.org/, accessed on 20 December 2024), were optimized by using the Discovery Studio 2.5 in the CHARMm force field and Generalized Born with Implicit Membrane(GBIM) for the Implicit Solvent Model. The minimized structures were then subjected to further evaluation using the Ramachandran plot. The 3D structure of baicalin (PubChem ID: 64982) was downloaded from the PubChem database (https://pubchem.ncbi.nlm.nih.gov/, accessed on 20 December 2024). The conformation of baicalin was optimized using OpenBabel [19] (https://openbabel.org/, accessed on 20 December 2024). Water molecules and ligands from the target proteins were removed using PyMOL (version 2.6). AutoDock Vina (version 1.5.7) software was used to perform molecular docking and calculate the binding energies between baicalin and the two proteins [20].

### 2.8. Statistical Analyses

The results from three independent experiments were subjected to one-way analysis of variance (ANOVA) and LSD multiple comparisons. *p* < 0.05 was considered statistically significant. All statistical analyses were performed using IBM SPSS Statistics 26, and all bar and line charts were created using GraphPad Prism 7.0 (GraphPad Software).

## 3. Results

### 3.1. Baicalin Decreased the Expressions of ROS in Serum and Inflammatory Factors in Jejunum Stimulated by LPS

**Figure 1:**
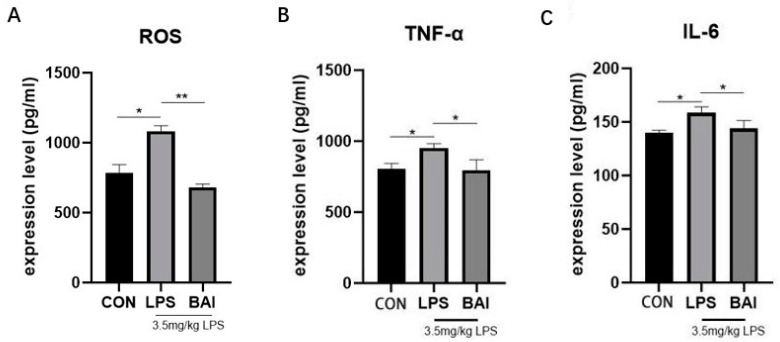
Effects of baicalin on LPS-induced inflammatory cytokines in *mice*. The effects of baicalin on ROS (**A**), TNF-α (**B**), and IL-6 (**C**) were determined by ELISA. The results indicate * *p* < 0.05, ** *p* < 0.01.

Compared to the control group, LPS significantly increased ROS levels in the serum (*p* < 0.05) (Figure 1A) and significantly elevated the expression levels of inflammatory factors TNF-α (*p* < 0.05) and IL-6 (*p* < 0.05) in the jejunum (Figure 1B,C), indicating that LPS treatment-induced oxidative stress and inflammation in the intestines of *mice*. Compared to the LPS group, baicalin significantly reduced ROS levels (*p* < 0.01) (Figure 1A) in the serum, as well as the expression of inflammatory cytokines TNF-α (*p* < 0.05) and IL-6 (*p* < 0.05) in the jejunum (Figure 1B,C), suggesting that baicalin effectively inhibited the secretion of these inflammatory cytokines and alleviated oxidative stress.

### 3.2. Effect of Baicalin on the Tissue Morphology of the Jejunum in Mice Induced by LPS

**Figure 2:**
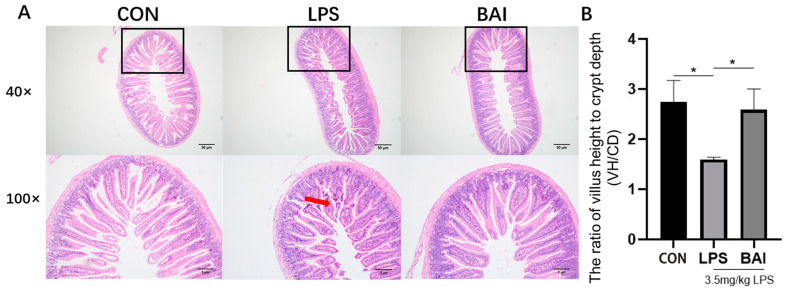
Effect of baicalin on histological changes in LPS-stimulated jejunum. Panel (**A**) represents H&E staining (up, scale bar = 50 μm; down, scale bar = 5 μm); Panel (**B**) shows the ratio of jejunum villus height to crypt. The red arrow indicates broken villi. * *p* < 0.05.

In Figure 2, jejunum tissue was processed for H.E. staining and examined under a microscope. As shown in the control group, the jejunum exhibited intact and orderly villous structures, with normal crypt structures and no significant hemorrhage or lymphoid tissue infiltration (Figure 2A). Compared to the control group, the jejunum in the model group was severely damaged, with broken villi and a significantly decreased villus-to-crypt ratio (Figure 2B) (*p* < 0.05). The histopathological sections of the jejunum from the baicalin-treated group (200 mg/kg) showed improved villus structure, with neatly distributed villi and better-defined crypts compared to the LPS-stimulated jejunum. The villus-to-crypt ratio significantly increased in the baicalin group (Figure 2B) (*p* < 0.05). These results indicated that baicalin pretreatment effectively improved jejunal structural damage.

### 3.3. Effects of Baicalin on Key Proteins in the MAPK Signaling Pathway in the Mice Jejunum Induced by LPS

**Figure 3:**
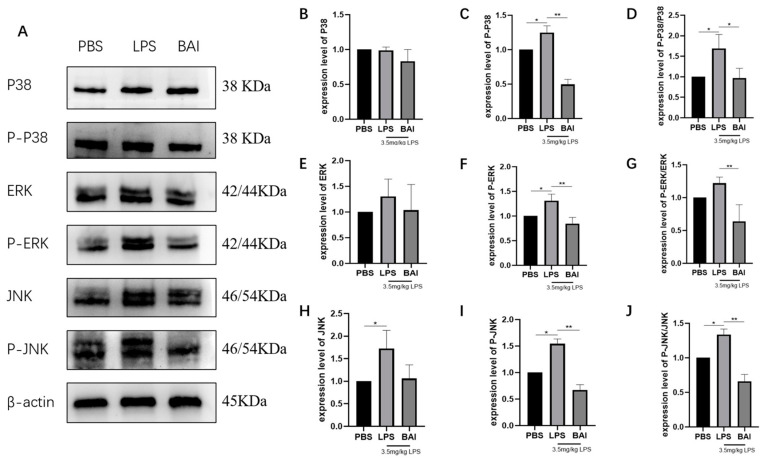
Effects of baicalin on key proteins of the MAPK signaling pathway in the jejunum. The levels of MAPK signaling pathway proteins (P38, p-P38, ERK, p-ERK, JNK, p-JNK, and β-actin) were determined by Western blot (**A**). P38 expression level (**B**), p-P38 expression level (**C**), p-P38/P38 expression level (**D**), ERK expression level (**E**), p-ERK expression level (**F**), p-ERK/ERK expression level (**G**), JNK expression level (**H**), p-JNK expression level (**I**), and p-JNK/JNK expression level (**J**). The results indicate * *p* < 0.05, ** *p* < 0.01.

LPS stimulation significantly increased the protein expression of p-P38 (Figure 3C, *p* < 0.05), p-ERK (Figure 3F, *p* < 0.05), JNK (Figure 3H, *p* < 0.05), and p-JNK (Figure 3I, *p* < 0.05), as well as the ratios of p-P38/P38 (Figure 3D, *p* < 0.05) and p-JNK/JNK (Figure 3I, *p* < 0.05). Compared to the LPS group, baicalin significantly inhibited the elevated protein levels of p-P38 (Figure 3C, *p* < 0.01), p-ERK (Figure 3F, *p* < 0.01), and p-JNK (Figure 3I, *p* < 0.01) in the jejunum. Additionally, the ratios of p-P38/P38 (Figure 3D, *p* < 0.05), p-ERK/ERK (Figure 3G, *p* < 0.01), and p-JNK/JNK (Figure 3J, *p* < 0.01) were significantly reduced in the baicalin group. These results suggested that baicalin inhibited the activation of the MAPK signaling pathway induced by LPS in the jejunum of *mice*.

### 3.4. ROS Scavenger (NAC) Reduces the Expression of Inflammatory Cytokines and ROS in LPS-Stimulated IPEC-J2 Cells

**Figure 4:**
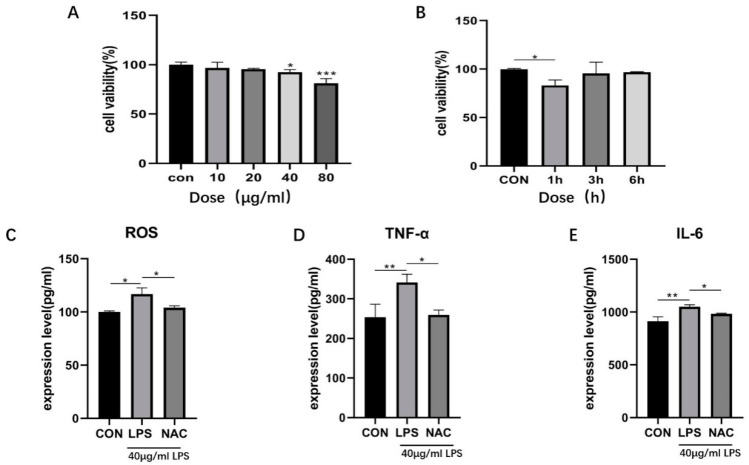
Establishment of the inflammatory model and the antioxidant effects of NAC. The CCK-8 method was used to determine the appropriate concentration (**A**) and time (**B**) for LPS treatment. The effect of NAC on ROS was measured by ELISA (**C**). The effects of NAC on LPS-stimulated inflammatory cytokines in IPEC-J2 cells were determined. Effects of NAC on TNF-α (**D**) and IL-6 (**E**) were assessed by ELISA. The results indicate * *p* < 0.05, ** *p* < 0.01, *** *p* < 0.001.

The appropriate time and concentration of LPS were selected based on changes in cell activity. LPS treatment for 1 h significantly reduced cell activity at concentrations of 40 μg/mL (Figure 4A, *p* < 0.05) and 80 μg/mL (Figure 4A, *p* < 0.001). At 40 μg/mL for 1 h, cell activity was significantly decreased (Figure 4B, *p* < 0.05), but no significant change was observed at 3 and 6 h. These results indicated that the cells developed tolerance to LPS treatment over time. Therefore, the optimal concentration and treatment time were determined to be 40 μg/mL and 1 h, respectively.

Compared to the control group, the expression levels of ROS (Figure 4C, *p* < 0.05), TNF-α (Figure 4D, *p* < 0.01), and IL-6 (Figure 4E, *p* < 0.01) in the LPS group were significantly increased. Compared to the LPS group, NAC significantly inhibited ROS production (Figure 4C, *p* < 0.05) and the expression levels of inflammatory factors TNF-α (Figure 4D, *p* < 0.05) and IL-6 (Figure 4E, *p* < 0.05). These results suggested that NAC reduced the expression of inflammatory cytokines (TNF-α and IL-6) in LPS-stimulated IPEC-J2 cells.

### 3.5. ROS Scavenger (NAC) Reduces the Expression of Key Proteins of the MAPK Signaling Pathway in LPS-Stimulated IPEC-J2 Cells

**Figure 5:**
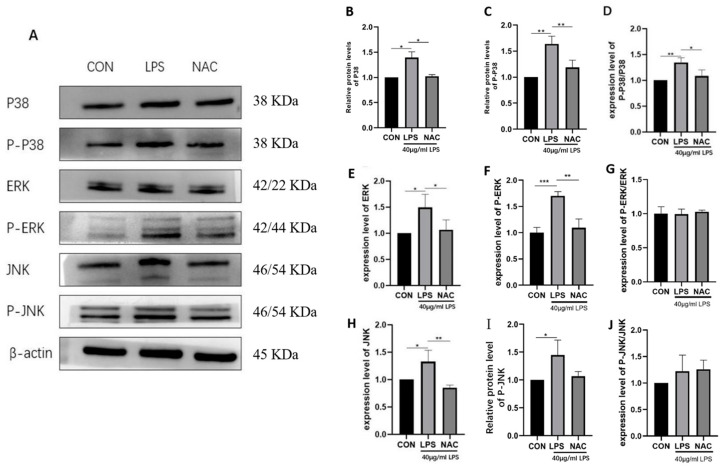
Effects of NAC on key proteins of the MAPK signaling pathway in LPS-stimulated IPEC-J2 cells. The protein expression levels of the MAPK signaling pathway proteins (P38, p-P38, ERK, JNK, p-JNK, p-P38/P38, and β-actin) were determined by Western blot (**A**). Expression levels of P38 (**B**), p-P38 (**C**), p-P38/P38 (**D**), ERK (**E**), p-ERK (**F**), p-ERK/ERK (**G**), JNK (**H**), p-JNK (**I**), and p-JNK/JNK (**J**). The results indicate * *p* < 0.05, ** *p* < 0.01, *** *p* < 0.001.

LPS significantly upregulated the protein levels of P38 (Figure 5B, *p* < 0.05), P-P38 (Figure 5C, *p* < 0.01), p-P38/P38 (Figure 5D, *p* < 0.01), ERK (Figure 5E, *p* < 0.05), p-ERK (Figure 5F, *p* < 0.001), JNK (Figure 5H, *p* < 0.05), and p-JNK (Figure 5I, *p* < 0.05). Compared to the LPS group, NAC significantly decreased the protein levels of P38 (Figure 5B, *p* < 0.05), P-P38 (Figure 5C, *p* < 0.01), p-P38/P38 (Figure 5D, *p* < 0.05), ERK (Figure 5E, *p* < 0.05), p-ERK (Figure 5F, *p* < 0.01), and JNK (Figure 5H, *p* < 0.01), suggesting that NAC reduced the expression of key proteins in the LPS-induced MAPK signaling pathway.

### 3.6. Baicalin Reduces the Expression of Inflammatory Factors, ROS and Lactate Dehydrogenase (LDH) in LPS-Stimulated IPEC-J2 Cells

**Figure 6:**
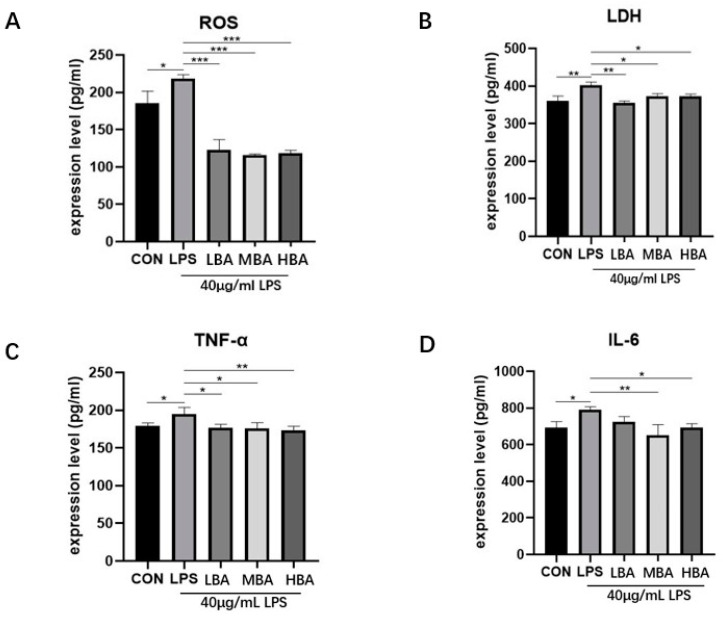
Effects of baicalin on oxidative stress-related proteins in LPS-stimulated IPEC-J2 cells, as determined by ELISA for ROS (**A**) and LDH (**B**). Effects of baicalin on LPS-stimulated inflammatory cytokines in IPEC-J2 cells, with TNF-α (**C**) and IL-6 (**D**) assessed by ELISA. The results indicate * *p* < 0.05, ** *p* < 0.01, *** *p* < 0.001.

Compared to the control group, the expression levels of ROS (Figure 6A, *p* < 0.05), LDH (Figure 6B, *p* < 0.01), and the inflammatory factors TNF-α (Figure 6C, *p* < 0.05) and IL-6 (Figure 6D, *p* < 0.05) in the LPS group were significantly increased. Baicalin concentrations were 10, 20, and 40 μM, as determined from previous results [21]. Compared to the LPS group, baicalin significantly inhibited the expression of ROS (Figure 6A, *p* < 0.001), LDH (Figure 6B, *p* < 0.05), and inflammatory factors TNF-α (Figure 6C, *p* < 0.05) and IL-6 (Figure 6D, *p* < 0.05). Notably, baicalin decreased the expression level of TNF-α in a dose-dependent manner. These results suggest that LPS treatment induces intestinal inflammation and oxidative stress in IPEC-J2 cells, while baicalin effectively inhibits the increase in inflammatory factors and the occurrence of oxidative stress.

### 3.7. Baicalin Reduces the Expression of Key Proteins of the MAPK Signaling Pathway in LPS-Stimulated IPEC Cells

**Figure 7:**
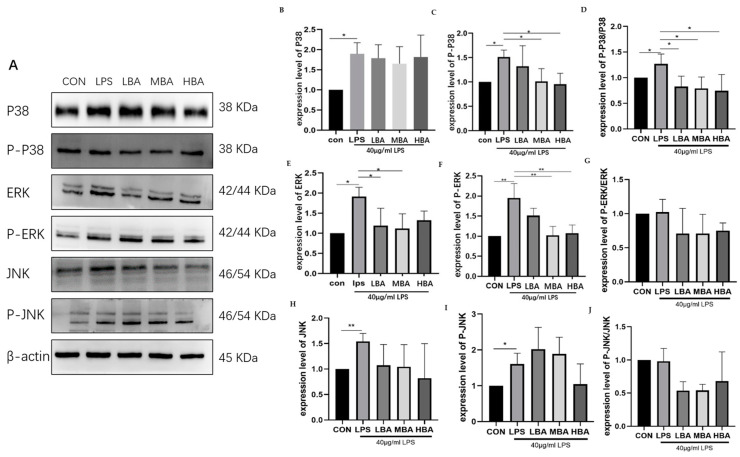
Effects of baicalin on key proteins of the MAPK signaling pathway in LPS-stimulated IPEC-J2 cells. The levels of MAPK signaling pathway proteins (ERK, p-ERK, P38, p-P38, p-P38/P38, JNK, p-JNK, and β-actin) were determined by Western blot (**A**). Expression levels of P38 (**B**), p-P38 (**C**), p-P38/P38 (**D**), ERK (**E**), p-ERK (**F**), p-ERK/ERK (**G**), JNK (**H**), p-JNK (**I**), and p-JNK/JNK (**J**). The results indicate * *p* < 0.05, ** *p* < 0.01.

Compared to the control group, LPS-stimulated IPEC-J2 cells exhibited significantly increased protein levels of P38 (Figure 7B, *p* < 0.05), p-P38 (Figure 7C, *p* < 0.05), p-P38/P38 (Figure 7D, *p* < 0.05), ERK (Figure 7E, *p* < 0.05), p-ERK (Figure 7F, *p* < 0.01), JNK (Figure 7H, *p* < 0.01), and p-JNK (Figure 7I, *p* < 0.05). Compared to the LPS group, baicalin at 10 μM significantly reduced the protein levels of p-P38/P38 (Figure 7D, *p* < 0.01) and ERK (Figure 7E, *p* < 0.05). Baicalin at 20 μM significantly inhibited the protein levels of p-P38 (Figure 7C, *p* < 0.05), p-P38/P38 (Figure 7D, *p* < 0.01), ERK (Figure 7E, *p* < 0.05), and p-ERK (Figure 7F, *p* < 0.01). Baicalin at 40 μM significantly inhibited the protein levels of p-P38 (Figure 7C, *p* < 0.05), p-P38/P38 (Figure 7D, *p* < 0.01), and p-ERK (Figure 7F, *p* < 0.01). Moreover, baicalin at 10, 20, and 40 μM significantly inhibited the ratio of p-P38/P38 (*p* < 0.05). However, baicalin did not significantly change the ratio of p-P38, p-ERK/ERK, JNK, p-JNK, or p-JNK/JNK. These results suggest that baicalin inhibits the expression of key proteins in the MAPK signaling pathway (p-P38, ERK, and p-ERK) in LPS-stimulated IPEC-J2 cells.

### 3.8. Molecular Docking of Baicalin Binding to Target Proteins

**Figure 8:**
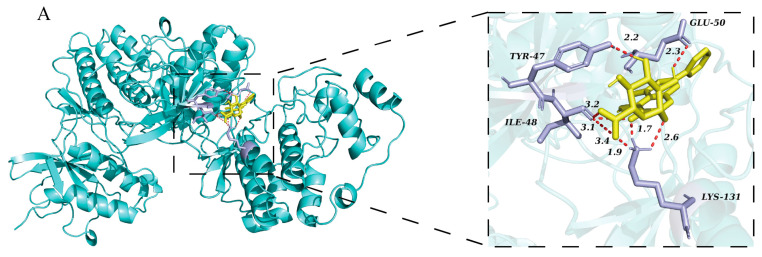

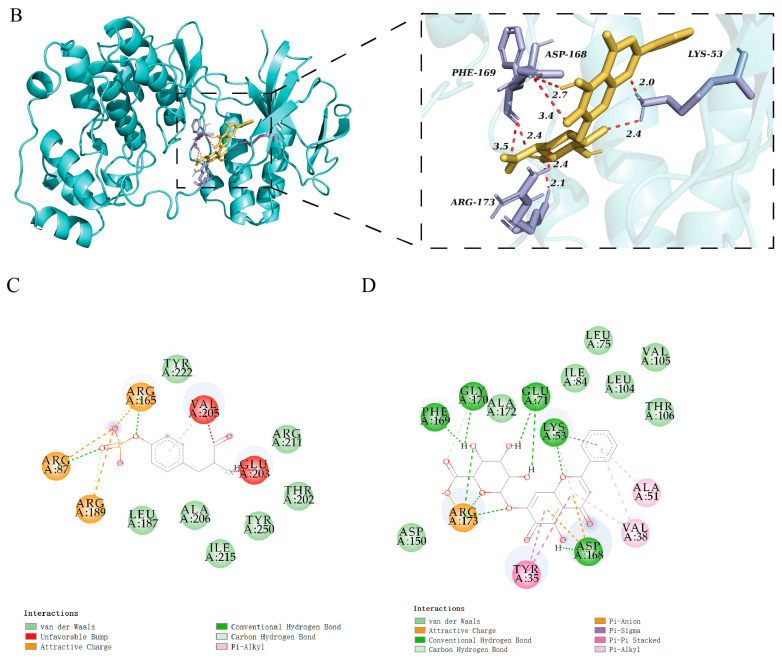
The 2D and 3D views of baicalin in complex with target proteins. (**A**) 3D view of baicalin (PubChem ID: 64982) in complex with ERK protein (PDB ID: 2zoq) after docking; (**B**) 3D view of baicalin (PubChem ID: 64982) in complex with P38 protein (PDB ID: 1bl7) after docking. (**C**) 2D view of baicalin (PubChem ID: 64982) in complex with ERK protein (PDB ID: 2zoq) after docking; (**D**) 2D view of baicalin (PubChem ID: 64982) in complex with P38 protein (PDB ID: 1bl7) after docking.

The minimized energies of ERK and P38 were found to be −40,932.13 kcal/mol and −20,050.40 kcal/mol, respectively, and the final RMS gradient values of ERK and P38 were determined to be 0.66 and 0.62, respectively. The results of the Ramachandran plots are provided in the Supplementary Files. The molecular docking results were visualized using PyMOL. Figure 8 presents the 2D and 3D views after docking. The binding energies of baicalin to ERK and P38 were −10.18 kcal/mol and −10.25 kcal/mol, respectively. These binding energies were superior to those of the ERK inhibitor 5IOD [22] (−8.03 kcal/mol) and the P38 inhibitor SB220025 [23] (−7.9 kcal/mol). The details are shown in Table 4.

## 4. Discussion

The practice of early weaning has been shown to induce considerable stress in *piglets*, which in turn has been observed to precipitate intestinal inflammation and diarrhea [24]. As a principal component of the cell wall of Escherichia coli, LPS can elicit a robust immune response in the host, resulting in intestinal inflammation [25]. In this study, LPS caused significant disruption to the intestinal structure in *mice*, as evidenced by increased protein expression of cytokines (TNF-α, IL-6) both in vivo and in vitro. This suggests that the LPS-induced inflammation models were successfully established. During the inflammatory process, oxidative stress frequently occurs concurrently, leading to elevated ROS levels and organelle damage [26]. LPS induces oxidative stress through the nuclear factor-erythrocyte 2-associated factor 2 (Nrf2) -heme oxidase-1 (HO-1) signaling pathway in IPEC-J2 cells [27,28]. Furthermore, LPS has been shown to elevate ROS levels in intestinal epithelial cells of rats and chickens [29]. LDH, a glycolytic enzyme widely distributed in various tissues and cells of the body, is released into the extracellular space following cell damage [30]. In this study, LPS damaged the jejunal structure and significantly increased ROS content in the serum of mice; in vitro, LPS also significantly increased LDH expression and ROS content, both indicating that LPS-induced oxidative stress in the organism. These models are stable and suitable for further research into the interaction between inflammation and oxidative stress.

As the upstream target of cytokines in the inflammatory response, MAPK signaling pathways represent a key point of intervention in the treatment of intestinal barrier damage induced by inflammatory or oxidative stress [31,32]. For example, berberine has been shown to enhance intestinal barrier function and mitigate inflammation and oxidative stress by modulating the NF-κB/MAPK signaling pathway in deoxynivalenol-challenged *piglets* [33]. ROS can participate in the activation of the MAPK signaling pathway, and the ROS/ERK/p38 signaling pathway has been shown to play a pro-inflammatory role in endothelial cells of the umbilical vein [34]. It was postulated that ROS/MAPK signaling pathways may serve as potential mediators of communication between inflammation and oxidative stress. In *mice*, the administration of LPS resulted in the activation of the JNK protein and the phosphorylation of MAPKs (P-ERK, P-JNK, and P-P38). In IPEC-J2 cells, the increased ROS content activated the MAPK proteins (ERK, JNK, and P38) and their phosphorylation (P-ERK, P-JNK, and P-P38) during LPS-induced inflammatory responses. The decreased ROS content induced by NAC significantly blocked the protein expressions of MAPKs (ERK, JNK, and P38) and their phosphorylation (P-ERK, P-JNK, and P-P38). Additionally, NAC was observed to decrease the expression of cytokines (TNF-α and IL-6). These results demonstrated that ROS/MAPK signaling pathways were indeed involved in LPS-induced inflammatory responses. Furthermore, the JNK, P-ERK, and P-P38 proteins were identified as common targets regulated by ROS in both in vivo and in vitro models.

**Figure 9:**
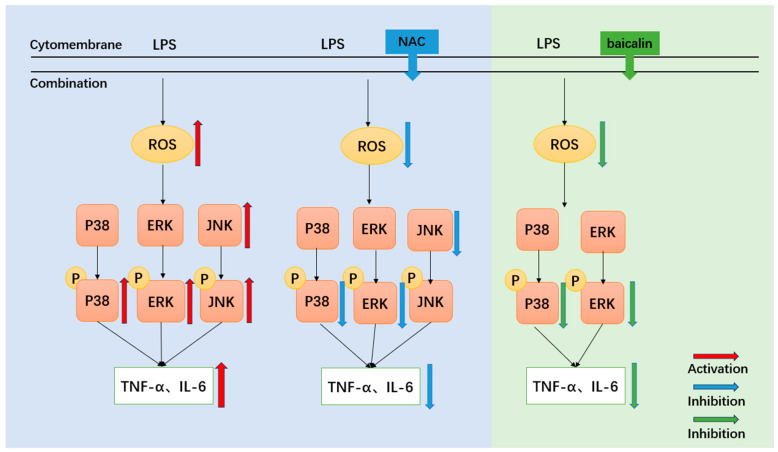
Baicalin can significantly reduce LPS-induced inflammation by ROS/p-ERK/p-P38 signal pathways.

It is known that baicalin can reduce inflammation both in *mice* and IPEC-J2 cells [35,36,37], and it can alleviate oxidative stress and inflammation in the treatment of diabetic nephropathy [34]. However, the impact of baicalin on the ROS/MAPK signaling pathways in the context of intestinal inflammation remained unclear. In *mice*, baicalin at a dose of 200 mg/kg significantly decreased ROS content, repaired the structure of the jejunum, and downregulated the expression levels of P-MAPKs (P-ERK, P-JNK, and P-P38). Additionally, baicalin reduced the secretion of cytokines (TNF-α, IL-6) in the jejunum. In IPEC-J2 cells, baicalin significantly decreased ROS content, downregulated the protein expression levels of P-ERK and P-P38, and inhibited the secretion of cytokines (TNF-α, IL-6) compared to the LPS group. It was observed that the P-ERK and P-P38 proteins, which are downstream targets of ROS, were the same targets regulated by baicalin in both in vivo and in vitro models. In silico studies of baicalin’s interactions with its molecular targets demonstrated that baicalin could effectively bind with ERK and P38 proteins. Our experimental data, obtained under real-world conditions, confirmed that baicalin blocked the phosphorylation process of ERK and P38 proteins. This indicates that baicalin alleviated inflammatory responses in vitro and in vivo by acting as a phosphorylation blocker of ERK and P38 proteins. Overall, baicalin can reduce inflammatory responses by regulating the ROS/p-ERK/p-P38 MAPK signaling pathway in vivo and in vitro (Figure 9).

In vitro, the typical dosage range of baicalin is 1–50 μM, particularly in RAW264.7 cells [35]. The baicalin dosage (10–40 μM) used in this manuscript was based on our previous screening range (4–16 μg/mL or 9.0–36.0 μM baicalin) [21]. The administration of baicalin for treating pathogenic bacterial infections in pigs and poultry is primarily achieved through continuous treatment for seven days at a dose of 50–200 mg/kg body weight [13]. In this manuscript, baicalin was administered orally at a dose of 200 mg/kg to defend against LPS-induced inflammatory responses in *mice*, which falls within the commonly used concentration range. This dosage range is comparable to that of quercetin (25 μM in vitro, 50 mg/kg in vivo) [38,39], but lower than that of forsythia suspension polyphenols (62.5–250 μg/mL in vitro, 200–600 mg/kg in vivo) in the treatment of enteritis [40]. In cases of severe infection, such as mycoplasma infection in chickens, the therapeutic dose may be increased to 450 mg/kg body weight [13]. A comparison of the data reveals that baicalin exhibits notable pharmacological effects and an excellent safety profile, offering promising prospects for its clinical use in the treatment of intestinal diseases. Furthermore, pharmacokinetic studies of baicalin can provide valuable insights into its potential for subsequent clinical trials. In Wistar rats intragastrically administered 200 mg/kg baicalin, the maximum plasma concentration reached 0.89 mg/L, the plasma half-life (T1/2) was 4.59 h, and the area under the curve of plasma concentrations was 12.23 mg/L·h (0–∞) [41]. However, there is a lack of the literature examining the toxicity or functional damage of vital organs resulting from baicalin exposure. The key points of this article are as follows: baicalin has the potential to be developed as a drug for the treatment of intestinal diseases such as UC or IBD; inflammation and oxidative reactions should be considered together when evaluating intestinal-related diseases, and the coordination of the ROS/p-ERK/p-P38 pathway in inflammation and oxidative reactions should be emphasized. Furthermore, investigating whether ROS/p-ERK/p-P38 plays a pivotal role in inflammasome formation or the development of inflammatory storms in the intestine would be beneficial.

## 5. Conclusions

Baicalin regulated inflammatory responses through the ROS/p-ERK/p-P38 signaling pathway in *mice* and IPEC-J2 cells, suggesting that inflammation and oxidative reactions should be considered together when evaluating piglet weaning stress. The coordination of the ROS/p-ERK/p-P38 pathway in inflammation and oxidative reactions should be emphasized. Future studies should investigate whether ROS/p-ERK/p-P38 plays a pivotal role in the development of inflammatory storms in the intestine.

## Figures and Tables

**Table 1:** Experimental grouping and baicalin management in vitro.

Grouping	Dispose
control group (CON)	1–7 d 200 μL PBS by gavage
LPS group (LPS)	1–7 d 200 μL PBS by gavage, 7 d 200 μL LPS intraperitoneal (3.5 mg kg^−1^)
Baicalin (BAI)	1–7d, BAI gavage for 7d (200 mg kg^−1^), 7 d 200 μL LPS intraperitoneal (3.5 mg kg^−1^)

**Table 2:** Experimental grouping and NAC management in vitro.

Grouping	Dispose
control group (CON)	No processing
LPS group (LPS)	LPS processing 1 h (40 μg/mL)
N-Acetyl-L-cysteine (NAC)	1 mL LPS treatment for 1 h (40 μg/mL) after pretreatment with 1 mL NAC (diluted 1:500)

**Table 3:** Experimental grouping and baicalin management in vitro.

Grouping	Dispose
control group (CON)	No processing
LPS group (LPS)	LPS processing 1 h (40 μg/mL)
Low-dose baicalin group (LBA)	1 mL baicalin pretreatment for 24 h (10 μM), 1 mL LPS treatment for 1 h (40 μg/mL)
Medium-dose baicalin group (MBA)	1 mL baicalin pretreatment for 24 h (20 μM), 1 mL LPS treatment for 1 h (40 μg/mL)
High-dose baicalin group (HBA)	1 mL baicalin pretreatment for 24 h (40 μM), 1 mL LPS treatment for 1 h (40 μg/mL)

**Table 4:** The center coordinates and molecular docking binding energy after analysis are as follows.

Protein (PDB ID)	Center Coordinate (XYZ)	Inhibitor (Binding Energy)	Baicalin Binding Energy
ERK (2ZOQ)	28.290769, 7.829962, 13.876654	5IOD (−8.03 kal/mol)	−10.18 kal/mol
P38 (1BL7)	2.279160, 12.465800, 29.790320	SB220025 (−7.9 kal/mol)	−10.25 kal/mol

## Data Availability

The datasets used and/or analyzed during the current study are available from the corresponding author on reasonable request.

## Supplementary Materials

The following supporting information can be downloaded at: https://www.mdpi.com/article/10.3390/biomedicines13020251/s1, Figure S1: The Ramachandran Plots of normal and optimized target proteins.

## List of Abbreviations

IPEC-J2 cells	porcine intestinal epithelial cells
LPS	Lipopolysaccharide
NAC	N-Acetyl-L-cysteine
BAI	Baicalin
MAPK	mitogen-activated protein kinase
ERK	extracellular regulated protein kinases
P38	P38 MAPK
JNK	c-Jun N-terminal kinase
P-ERK	*Phospho-* extracellular regulated protein kinases
P-P38	*Phospho-P38 MAPK*
P-JNK	*Phospho-* c-Jun N-terminal kinase
ROS	reactive oxygen species
LDH	Lactate dehydrogenase
TNF-α	tumor necrosis factor
IL-6	Interleukin-6
BCA	bicinchoninic acid
ECL	enhanced chemiluminescence
