# Supplementary material for: Baicalin Decreases the LPS-Induced Intestine Inflammatory Responses by ROS/p-ERK/p-P38 Signal Pathways In Vivo and In Vitro
Source: Biomedicines. 2025 Jan 21;13(2):251. doi: 10.3390/biomedicines13020251 (PMC11852140; doi:10.3390/biomedicines13020251)
Supplement: Supplementary file 1 [file biomedicines-13-00251-s001.zip › biomedicines-3356047-supplementary.pdf]

The proportion of residues in most favored regions in the optimized ERKstructure was 83.8%, which was better than that of the crystal structure (81.7%) (Fig.S1 A and B). Meanwhile, the proportions of residues in most favored regions for minimized and un-minimized P38 structures were 89.9% and 89.8%, respectively (Fig.S1 C and D).

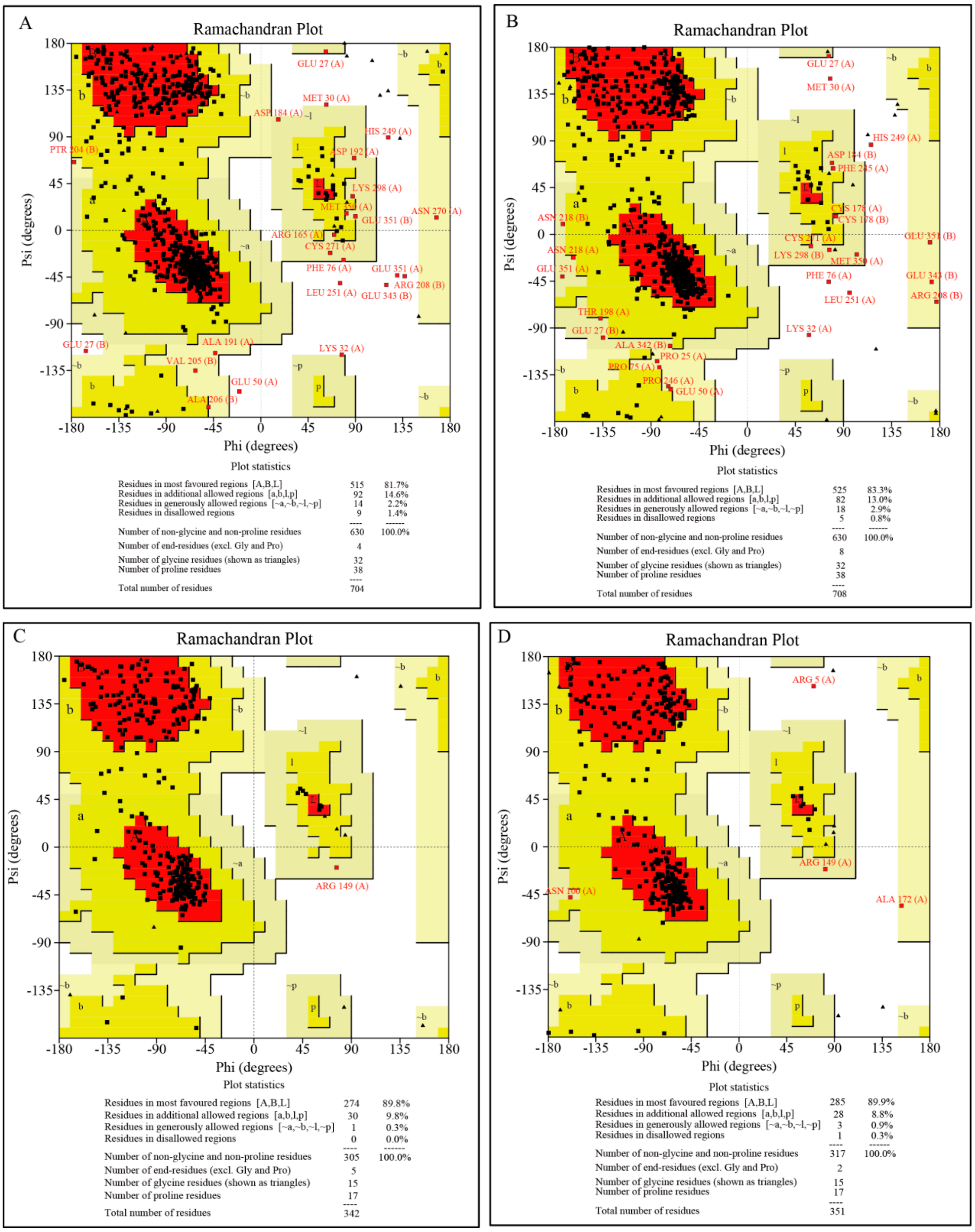

Fig.S1 (A) ERK (PDB ID: 2zoq) protein Ramachandran Plot; (B) Optimized ERK (PDB ID: 2zoq) protein Ramachandran Plot; (C) P38 (PDB ID: 1bl7) protein

Ramachandran Plot; (D) Optimized P38 (PDB ID: 1bl7) protein Ramachandran Plot.
